# Unraveling the Microbial Symphony: Impact of Antibiotics and Probiotics on Infant Gut Ecology and Antibiotic Resistance in the First Six Months of Life

**DOI:** 10.3390/antibiotics13070602

**Published:** 2024-06-27

**Authors:** Qi Qi, Liang Wang, Yingze Zhu, Shaoru Li, Mitslal Abrha Gebremedhin, Baozhu Wang, Zhonghai Zhu, Lingxia Zeng

**Affiliations:** 1 Department of Epidemiology and Biostatistics, School of Public Health, Xi’an Jiaotong University Health Science Center, Xi’an 710061, China; kikistronger@stu.xjtu.edu.cn (Q.Q.); wl673896343@stu.xjtu.edu.cn (L.W.); zhuyingze@stu.xjtu.edu.cn (Y.Z.); mitslal2016@stu.xjtu.edu.cn (M.A.G.); baozhu_only@163.com (B.W.); 2 Experimental Teaching Center, School of Public Health, Xi’an Jiaotong University Health Science Center, Xi’an 710061, China; lishaoru@xjtu.edu.cn; 3 Department of Health, Northwest Women’s and Children’s Hospital, Xi’an 710003, China; 4 Center for Chronic Disease Control and Prevention, Global Health Institution, Xi’an Jiaotong University, Xi’an 710061, China; 5 Key Laboratory for Disease Prevention and Control and Health Promotion of Shaanxi Province, Xi’an 710061, China

**Keywords:** infant, antibiotics, probiotics, gut microbiota, antibiotic resistance, early infancy, delivery mode

## Abstract

We aimed to examine the effects of antibiotic and probiotic usage on the gut microbiota structure and the presence of antibiotic-resistance genes (ARGs) in infants during the first six months of life. Questionnaires and fecal samples were collected within three days of birth, two months, and six months to assess antibiotic and probiotic exposure. Gut microbiotas were sequenced via 16S rRNA, and ARGs were conducted by qPCR, including beta-lactam (*mecA*, *bla*_TEM_), tetracycline (*tetM*), fluoroquinolone (*qnrS*), aminoglycoside (*aac(6′)-Ib*), and macrolide (*ermB*). Infants were categorized by antibiotic and probiotic usage and stratified by delivery mode, microbial composition, and ARG abundances were compared, and potential correlations were explored. A total of 189 fecal samples were analyzed in this study. The gut microbiota diversity (Chao1 index) was significantly lower in the “only probiotics” (PRO) group compared to the “neither antibiotics nor probiotics” (CON) group at six months for the CS stratification (*p* = 0.029). Compositionally, the abundance of core genus *Bifidobacterium_pseudocatenulatum* was less abundant for the antibiotic during delivery (IAP) group than that in the CON group within the first three days (*p* = 0.009), while core genus *Enterococcus_faecium* was more abundant in the PRO than that in the CON group (*p* = 0.021) at two months. ARGs were highly detected, with *Enterococcus* hosting *tetM* and *Escherichia* associated with *bla*_TEM_ within three days of birth, though no correlation was found between *Bifidobacterium* and ARGs. These findings emphasized the critical importance of carefully managing antibiotic and probiotic exposures in early life, with implications for promoting lifelong health through preserving a healthy infant gut ecosystem.

## 1. Introduction

Gut microbiota plays a crucial role in infant health and development. During the first six months of life, the gut microbiota undergoes highly dynamic changes, reaching a stable, adult-like state by 2–3 years of age [1,2]. Disturbances to early life gut microbiota have been associated with increased risks of chronic conditions, including obesity, metabolic syndrome, diabetes, and immune system disorders [3,4,5]. As such, promoting healthy gut microbiota development in infancy may be crucial for lifelong well-being. Researchers have increasingly focused on how antibiotics and probiotics impact the infant’s gut microbiota [6,7]. Understanding these interventions may provide valuable insights for mitigating disease risks in childhood and adulthood.

Antibiotics are commonly prescribed to infants to treat bacterial infections, beginning at the time of delivery. Specifically, intrapartum antibiotic prophylaxis (IAP) is a clinical measure used in more than 30% of deliveries to prevent group B Streptococcus (GBS) infection that may be administered before or during a cesarean section (CS) to prevent infection [8,9]. However, researchers have revealed adverse effects of IAP, including delayed microbial maturation and altered microbiota establishment [10,11]. Furthermore, children are highly prescribed antibiotics in China (43.5%) at a rate higher than those reported in many countries, such as France (26.1%) and Australia (23%) [12]. Antibiotic exposure during early life has been linked to disrupted microbial colonization, decreased microbial diversity and stability, increased risk of gut dysbiosis and chronic diseases, and elevated levels of antibiotic resistance genes (ARGs) [13,14,15].

Studies have shown that probiotics have potential significance in modulating and clinically counteracting antibiotics’ adverse effects on the gut microbiota [16,17]. However, probiotic co-prescription rates remain low, particularly in the Asia–Pacific region [18]. Controlled trials in infants have shown that probiotic interventions increase the relative abundance of *Bifidobacterium infantis* and *Lactobacillus*, restore microbial diversity, decrease the relative abundance of pathogenic commensal bacteria, and reduce antibiotic resistance in gut microbiota, indicating better restoration of the child [17,19,20]. Yet, the efficacy of probiotics in treating microbiota dysbiosis in infants varies in clinical practice. Specific probiotic strains have demonstrated no significant benefit over the placebo in eradicating antibiotic-resistant colonization, and probiotic genome analyses have identified potential risks of spreading antibiotic resistance [21,22].

Previous studies have often examined antibiotics and probiotics separately, using cross-sectional or retrospective designs with limited samples and durations. This highlights the need for research on their combined effects on gut microbiota and antibiotic resistance in infants under six months, particularly in rural areas with high rates of antibiotic misuse. This study aimed to investigate the distinct impacts of antibiotic and probiotic administration on the composition and structure of gut microbiota and the prevalence of six commonly used ARGs in infants within the initial six months of life based on a well-characterized birth cohort.

## 2. Results

### 2.1. Sociodemographic Information of Mothers and Infants

A total of 77 newborns were enrolled in our study; of these, 55 and 57 infants were followed up at two and six months of age, respectively, so that 189 fecal samples were ultimately analyzed. A total of 70.1% of the neonates were delivered vaginally. During delivery, 23.4% of the neonates’ mothers used IAP, but none used probiotics. The usage of antibiotics and probiotics by infants in the first three days, at two months, and at six months is shown in Table 1. We defined the group that used antibiotics during delivery as IAP, those infants who used only antibiotics as ABX, those who used only probiotics as PRO, those who used both antibiotics and probiotics as ABX + PRO, and those who used neither antibiotics nor probiotics as CON.

### 2.2. Overview of Microbial Communities in Infants

After quality control and denoising procedures were carried out, the sequencing process resulted in a total of 13,197,758 high-quality reads with eligible sequence lengths. The number of reads per sequenced sample ranged from 38,802 to 84,787, with an average of 69,829 (SD 10,700). These reads were subsequently classified into 821 amplicon sequence variants (ASVs), which were used for further analysis.

**Figure 1:**
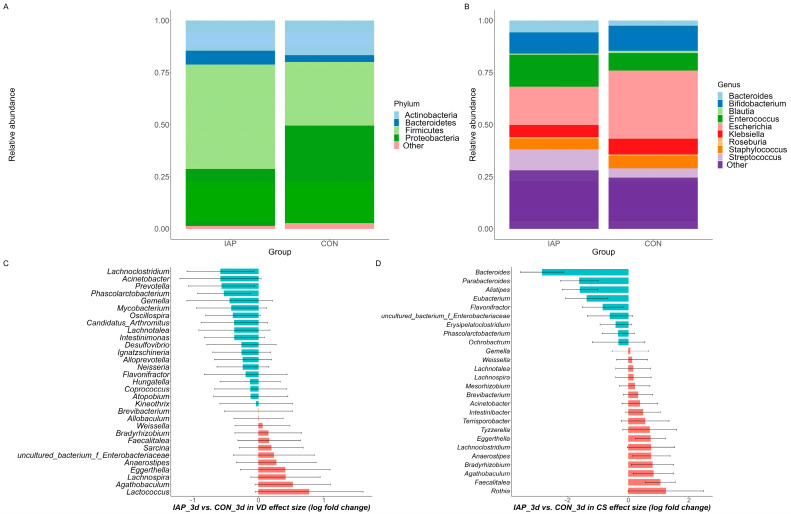
Relative abundance of gut microbial communities at the phylum level (**A**) and genus level (**B**) in the first three days. Waterfall plot of differentially abundant genus in the neonate microbiota derived from the ANCOM-BC model, representing beta values (log-fold change) by (**C**) vaginal delivery (VD) and (**D**) cesarean section (CS) after birth. The X-axis represents the log-fold change in beta values in the differential abundance of taxa in the IAP group versus the CON group, while the Y-axis represents differentially abundant taxa at the genus level. All effect sizes were adjusted by the Bonferroni method (*q* < 0.05). Taxa represented by blue bars are abundant in the IAP group, while those represented by red bars are abundant in the CON group. Statistical significance was determined at the *p* < 0.05 and *p* < 0.001 levels. VD, vaginal delivery; CS, cesarean section; IAP, intrapartum antibiotic prophylaxis; CON, use neither antibiotics nor probiotics.

To gain a clear picture of communities of infant gut microbiota, we carried out analyses at the phylum and genus levels. We found that the phyla Actinobacteria, Bacteroidetes, Firmicutes, and Proteobacteria were present at levels exceeding 0.01% in at least 75% of the samples taken in the first three days of life. In both stratification groups, Firmicutes and Proteobacteria were the dominant phyla, comprising over 80% of all phyla present (Figure 1A). At the genus level, we identified and filtered out rare species whose relative abundance was less than 0.01% while retaining species that were present in at least 80% of samples, as shown in Figure 1B. ANCOM-BC results revealed that, in the vaginal-delivery stratification, genera such as *Lachnoclostridium*, *Acinetobacter*, and *Prevotella* exhibited upregulation in the IAP group, while *Lachnospira*, *Agathobaculum*, and *Lactococcus* showed upregulation in the CON group. In the cesarean section stratification, *Bacteroides*, *Parabacteroides*, and *Alistipes* were the top three genera upregulated in the IAP group, while *Agathobaculum*, *Faecalitalea*, and *Rothia* were the top three genera upregulated in the CON group (Figure 1C,D). The complete list is presented in Supplementary Table S1. Correspondingly, microbial community compositions at two months and six months of age are shown in Supplementary Figure S1.

### 2.3. Impact of Antibiotics and Probiotics on Diversity of Gut Microbiota in Infants

**Figure 2:**
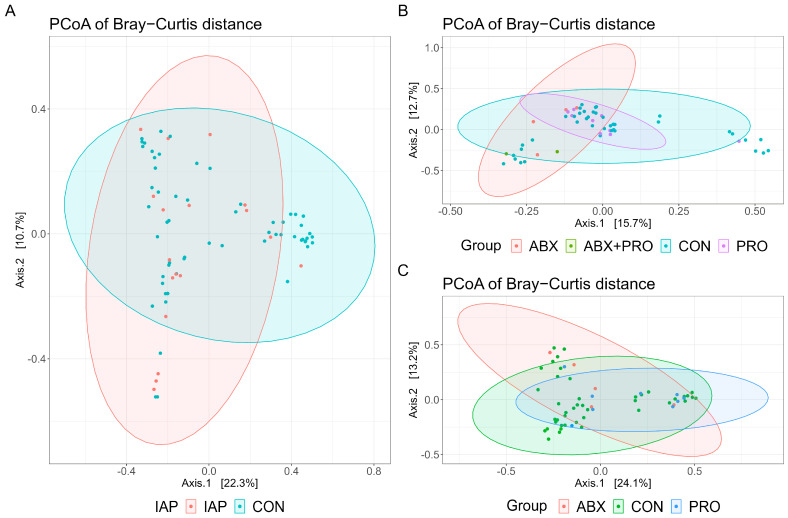
PCoA analysis of Bray–Curtis distance regarding the difference in the microbial community composition in the first three days (**A**), at two months (**B**), and at six months (**C**) of age (PERMANOVA with 9999 permutations, all *p* > 0.05). PERMANOVA, permutational multivariate analysis of variance.

We found no significant differences in the alpha diversities of gut microbiota in infants aged within three days or in those aged two months, with or without stratification (Table 2 and Table 3). For infants aged six months in the CS stratification, the Chao1 index showed a significant difference among groups (*p* = 0.049), and in the PRO group was significantly lower than that in the CON group (*p* = 0.029), see Table 3. However, there were no significant differences in the beta diversity of the microbial community structure among the groups of infants at any of the three time points (Figure 2, all *p* > 0.05). The results of the permutational multivariate analysis of variance (PERMANOVA) are shown in Table S2.

### 2.4. Core Genus Differences Resulting from Usage of Antibiotics and Probiotics

The analysis of 77 neonatal fecal samples identified 6 core gut microbiota genera, with *Escherichia_coli* as the dominant taxon, followed by *Bifidobacterium*_*pseudocatenulatum* and *Bifidobacterium_longum* (Table 4). Notably, the relative abundance of *Bifidobacterium_pseudocatenulatum* (ASV2) in the IAP group was significantly lower than that in the CON group (*p* = 0.009), which was the same as the CS stratification (*p* = 0.035). In samples taken from two-month-old infants, the ABX + PRO group was not shown due to the small sample size (*n* = 2). The abundance of *Enterococcus_faecium* (ASV6) was found to differ significantly among groups (*p* = 0.008), and the abundance in the PRO group was significantly higher than in the CON group (*p* = 0.021), which remained the same in the VD stratification (*p* = 0.019) (Table 5). In samples taken from infants at six months, the abundances of *Bifidobacterium_pseudocatenulatum* (ASV2), *Blautia_obeum* (ASV9), and *Erysipelatoclostridium_ramosum* (ASV34) were found to differ significantly among the groups (*p* = 0.004, *p* = 0.013, *p* = 0.026, respectively) (Table 6). After pairwise comparisons, it was found that the abundances of the three genera in the PRO group were significantly lower than that in the CON group (*p* = 0.002, *p* = 0.007, *p* = 0.011, respectively), which remained the same after stratification.

### 2.5. Effects of Antibiotic and Probiotic Use on Antibiotic Resistance Genes

As is shown in Table S3, the *aac(6′)-Ib* gene was detected in all samples, but the *ermB* and *tetM* genes were only detected at two months and six months, while the *qnrS* gene was only detected at six months. Overall, the detection rate of ARGs was high. In the unstratified analysis, no statistically significant differences in ARGs were observed among the groups. However, after stratification, in samples taken during the first three days of life, there was a higher trend in the relative abundance of the *qnrS* gene in the IAP subgroup (*p* = 0.052) and a lower trend in the relative abundance of the *bla*_TEM_ gene in the IAP subgroup compared with the CON group (*p* = 0.051). At six months, there were significant differences in the absolute abundances of *aac(6′)-Ib* and *tetM* genes among the groups of infants born vaginally (*p* = 0.057, *p* = 0.059). Pairwise comparisons between groups revealed that the relative abundance of the *aac(6′)-Ib* gene was lower in the ABX group compared with the CON group (*p* = 0.008), while the relative abundance of the *tetM* gene was higher in the ABX group, compared with the CON group (*p* = 0.013).

### 2.6. Correlation of Antibiotic Resistance Gens with Gut Microbiota Communities

**Figure 3:**
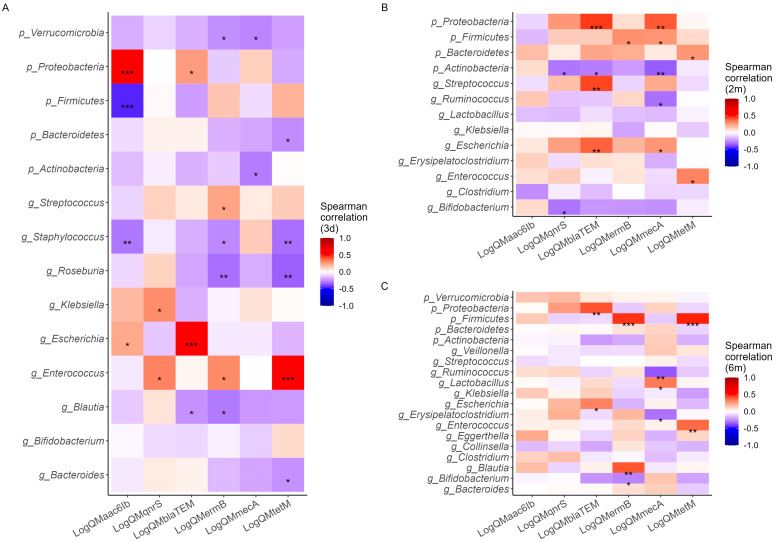
Correlation heatmaps between the log quality mean value of six ARGs and top microbiota at the phylum and genus level in the first three days (**A**), at two months (**B**), and at six months of age (**C**). The R–value is depicted using distinct colors. Additionally, the significance level is denoted by asterisks (*, **, and ***) to indicate that the correlation is significant at the 0.05, 0.01, and 0.001 levels, respectively.

A correlation heatmap was used to identify the specific microbial hosts of the ARGs (Figure 3). In infants within three days of age, the abundances of *aac(6′)-Ib* were significantly correlated with the phyla Proteobacteria and Firmicutes, suggesting that these taxa may serve as potential hosts for the *aac(6′)-Ib* gene. Similarly, the genus *Enterococcus* was identified as a potential host for *tetM*, while the genus *Escherichia* was associated with the *bla*_TEM_ gene. Notably, no significant correlation was observed between the genus *Bifidobacterium* and the ARGs examined. Correspondingly, at two months, the phylum Proteobacteria was associated with the *bla*_TEM_ gene, while at six months, the phylum Firmicutes was associated with the *ermB* and *tetM* genes. R-values and *p*-values are shown in Tables S4–S6.

## 3. Discussion

In this study, we characterized the composition of gut microbiota and the presence of ARGs in infants at three time points in the first six months of life. Due to loss to follow-up at two and six months of age, lost respondents were distributed across antibiotics and probiotics use groups; they were not statistically different from follow-up respondents in terms of mother and infant characteristics and, thus, would have no impact on the results. By exploring correlations between gut microbial communities, ARGs, and exposure to antibiotics and probiotics, we sought to elucidate potential interactions among these factors. Our findings revealed that the use of antibiotics and probiotics can significantly impact the core genera, and while antibiotic exposure was associated with altered ARG abundances, probiotic intake did not exhibit such effects. Additionally, we were able to identify putative microbial hosts for specific ARGs. These results enhance our understanding of the complex interplay between clinical interventions, the developing gut microbiota, and the emergence of antimicrobial resistance in early life, providing insights to guide evidence-based strategies for optimizing infant gut health and mitigating resistance risks.

The postnatal colonization and assembly of the infant gut microbiota is a highly dynamic process, and antibiotic exposure during this critical window can disrupt gut homeostasis, leading to the depletion of keystone taxa, diminished taxonomic diversity, altered metabolic functions, and the potential proliferation of pathogenic organisms [23]. In the present study, the predominant phyla in infants were found to be Actinobacteria, Bacteroidetes, Firmicutes, and Proteobacteria, which mirrored compositions reported previously [24]. Interestingly, further stratification by delivery mode revealed an upregulation of the genus *Lactococcus* associated with the CON group under the VD subgroup. Notably, a previous study reported a *Lactococcus* strain isolated from the maternal vagina that exhibited probiotic properties [25], which implies that antibiotic exposure during delivery may disrupt the vertical transmission of beneficial taxa like *Lactococcus* from the maternal to infant gut microbiota. We did not observe notable disparities in alpha and beta diversity in the first three days of life or at the two-month time point. This may be attributable to the uniform colonization and development characteristics of this early period. Alternatively, the effects of antibiotics or probiotics may not yet be manifested in such young infants, as prior research has demonstrated limited impacts of early-life probiotic exposure on gut microbiota diversity [26,27]. The results of the present study align with these previous findings.

Core microbiome genera are widely distributed and abundant across samples. Consistent with prior research [28,29,30], we found that exposure to IAP reduced the abundance of the core genus *Bifidobacterium* in infants within three days of age. Conversely, in healthy full-term infants without IAP exposure, *Bifidobacterium* dominated the gut. Furthermore, IAP has been shown to decrease *Bifidobacterium* over time while increasing opportunistic pathogens like *Clostridium difficile* [10,31]. This *Bifidobacterium* depletion may promote gut dysbiosis, including an elevated pH and proliferation of spore-forming bacteria [32]. Importantly, we observed a higher *Enterococcus_faecium* abundance in the probiotic-supplemented group at two months. As *Enterococcus* is commonly used clinically as a probiotic [33], this likely reflects the exogenous supplementation. By four to six months, as solid foods are introduced, genera like *Helicobacter* and *Clostridium* are typically established [34,35]. Interestingly, probiotic intake may competitively inhibit or modulate the gut environment to reduce these genera. Further mechanistic research is warranted to fully elucidate probiotic impacts on the infant gut microbiota.

The present study found a high prevalence of antibiotic resistance genes (ARGs) in the infant gut microbiota, which is consistent with previous longitudinal research detecting ARGs for aminoglycosides, beta-lactams, macrolides, and tetracyclines across the first year of life [36]. These ARG variations may stem from resistant bacterial strains present harboring specific ARGs that can spread to other strains. Additionally, infant exposure to environmental antibiotic residues may enhance the competitive advantage of resistant microbes. Notably, we did not find evidence that probiotic supplementation reduced ARG abundance, aligning with prior reports [37]. This may be explained by the variable antibacterial properties and intrinsic resistance profiles of different probiotic strains [38,39,40]. Thus, when selecting probiotics, it is critical to not only identify the species but also characterize their resistance determinants.

Previous work has postulated that ARGs could potentially find host organisms within microbial communities based on significant correlations between ARG genes and similarities of abundances, which were observed across various samples (*p* < 0.01; *r* > 0.6) [41]. Consistent with this, we observed associations between ARGs and the gut microbiota in the present study, suggesting that the microbiota harbors antibiotic resistance. In recent years, correlation analysis has been widely conducted to infer ARG hosts in fecal samples [42]. To build on these observations, future research should integrate sequencing technologies with functional metagenomics or genome assembly approaches to further validate ARG–microbe linkages.

In summary, the present study provides preliminary evidence linking antibiotics, probiotics, gut microbiota, and ARGs. This finding may be particularly relevant in regions with high antibiotic usage, highlighting the need for the appropriate regulation of these interventions. The longitudinal design enabled the observation and analysis of the microbial composition and antibiotic resistance within the first six months of infant life. The comprehensive dataset, including high-throughput sequencing, usage records, and resistance gene detection, offered insights into the effects of antibiotics and probiotics on the gut microbiota. However, there were some limitations to our study. First, the infant numbers in the antibiotics and probiotics groups were relatively small, and the group that used both antibiotics and probiotics was not shown due to the limited number in the follow-up samples. It is necessary to increase the number of findings to provide insights for future studies with larger sample sizes to elucidate the effects of combining antibiotics and probiotics for intervention. Second, qPCR methods have limitations compared to metagenomic sequencing for characterizing the resistome and microbiota, but this targeted and cost-effective technique provides insights into our future utilization of metagenomic sequencing to determine antibiotic resistance profiles. Third, though infants within six months have a relatively simple environment, potential confounding factors in the study may not be adequately controlled and can affect the interpretation of results, so it is difficult to establish causality. Furthermore, this study considered the cross-sectional exposure of antibiotics and probiotics without residual effects alongside the consideration of earlier exposures, which need to be analyzed further in a larger sample. Lastly, according to the WHO classification, combining Access (penicillins and cefazolin) and Watch (cefotiam) antibiotics in the analysis led to different attributable risks due to neglecting the prevalence of usage. Specifying the AWaRe category and analyzing it separately is crucial to highlight their appropriate use and public health significance. Therefore, further research is needed to better elucidate the mechanisms by which antibiotics and probiotics shape the developing gut microbiota and antibiotic resistance, emphasizing their public health implications.

## 4. Materials and Methods

### 4.1. Study Design and Participant Enrollment

This study was based on a birth cohort study conducted in northwest China from January 2018 to June 2019. Comprehensive details regarding the cohort are described elsewhere [43]. First, we collected 77 neonatal fecal samples; then, during follow-up visits at two months and six months of age, we collected 55 and 57 samples, respectively, due to loss of contact or the parents going out of town for work. IAP is recommended within 30-60 min prior to skin incision, with no difference in reducing the incidence of infectious morbidity after CS for single and multiple-dose regimens, but there are concerns about transmitting antibiotics and promoting antibiotic resistance in neonates via the umbilical cord [44]. Some providers withhold antimicrobials until after cord clamping. We considered IAP exposure as receiving intravenous antimicrobials during labor [45] and no-IAP exposure as no antimicrobial exposure, emergency CS, or exposure only after delivery. Follow-up antibiotic or probiotic exposure was assessed within seven days prior to sample collection. In total, 189 fecal samples were analyzed for microbiome composition and ARGs.

The inclusion criteria were as follows: (1) women with a full-term singleton fetus; (2) infants with complete antibiotic and probiotic records of delivery and follow-up visits; (3) and women who did not have any diagnosed gestational complications. The exclusion criteria covered women who could not complete the required investigation and follow-up visits.

During recruitment, the women were provided with a comprehensive explanation of the study and oral and written consent was obtained from all participants. The study received approval from the Institutional Review Board (IRB) of Xi’an Jiaotong University Health Science Center (No. 2018-293).

### 4.2. Collection of Sociodemographic Information

Birth outcome data, including maternal antibiotic and probiotic use, were obtained from hospital records. During follow-up visits, questionnaires collected information on sociodemographics, feeding patterns (including nutritional supplements and addition of complementary foods), disease and treatment history (focusing on the use of antibiotics and probiotics), and maternal medication use during breastfeeding. Maternal exposure to antibiotics during the perinatal period predominantly included cephalosporins and penicillin, with an initial dose of 3.2 million units of penicillin every 12 h or cefazolin/cefotiam every 6 h. Common reasons for infant antibiotic use included upper respiratory tract infections, pneumonia, and abdominal pain, with penicillin and cephalosporins as the predominant medications, and very few used macrolide antibiotics. According to the WHO AWaRe classification [46], penicillin and cefazolin are Access antibiotics, while cefotiam is a Watch antibiotic. However, due to sample size limitations, we conducted a combined analysis based on their similar mechanisms of action despite differences in their antibiotic accessibility. Moreover, probiotic products containing Bacillus, Bifidobacterium, Lactobacillus, and Enterococcus were commonly used.

### 4.3. Collection of Fecal Samples

After delivery, neonate fecal samples were obtained from diapers, either by parents or investigators, within the first three days of the infant’s life. Most of the samples were from either the first or second defecation. At follow-up time points, fecal samples were collected by parents and investigators and notified within 8 h so that samples could be collected. To ensure proper preservation, the fecal samples were carefully packaged, labeled, and promptly stored in a −20 °C refrigerator for short-term storage. Later, they were transferred to the laboratory and stored in a −80 °C freezer until further analysis.

### 4.4. DNA Extraction and High-Throughput 16s rRNA Gene Amplicon Sequencing

The gut microbiota composition was determined by means of the high-throughput sequencing of 16S rRNA gene amplicons. PCR amplification was carried out using barcoded bacterial primers (341F: 5′-CCTAYGGGRBGCASCAG-3′ and 806R: 5′-GGACTACNNGGGTATCTAAT-3′ [47]), targeting the V3-V4 variable region of the 16S rRNA gene. Then, a standard protocol was followed to generate sequencing libraries alongside paired-end reads using an Illumina HiSeq 2500 platform provided by Biomarker Technologies Co., Ltd. (Beijing, China). The sequencing platform generated FASTQ format files containing read information and sequence quality. Next, the raw data were processed; this involved filtering using Trimmomatic v0.33 [48] and the removal of primer sequences using cutadapt 1.9.1 [49] so that high-quality reads without primers were obtained. Denoising and the removal of chimeric sequences were then carried out using DATA2 [50] in QIIME 2020.6 [51], resulting in non-chimeric reads. Silva 16S rRNA was used to conduct taxonomic annotations of feature sequences [52].

### 4.5. Detection of Antibiotic-Resistant Genes Using qPCR

The study utilized cost-effective quantitative PCR (qPCR) analysis to quantify a panel of six clinically relevant antibiotic resistance genes (ARGs) across different antibiotic classes, reflecting both the environmental and antibiotic exposures seen in rural and urban infant populations [53,54]. The ARGs included beta-lactam (*mecA*, *bla*_TEM_), tetracycline (*tetM*), fluoroquinolone (*qnrS*), aminoglycoside (*aac(6′)-Ib*), and macrolide (*ermB*). Each sample was analyzed with three replicates for data accuracy.

### 4.6. Bioinformatic and Statistical Analysis

Statistical analysis and visualization were conducted using Stata 15.0 (Stata Corp., College Station, TX, USA) and R (version 4.1.0). Normally distributed continuous data were reported as the mean ± standard deviation, while non-normally distributed data were presented as the median and quartile (P25, P75). Count data were expressed as frequency (n) and percentage (%). Group comparisons were performed using the Chi-square test (χ2) or Fisher’s exact test for categorical variables. The significance level was defined as *p* < 0.05.

For microbiota analysis in R, the Phyloseq package [55] was employed to create an object comprising the ASV table, sample variables, and a taxonomy table. Then, we retained the sequencing depth of each sample without rarefaction [56]. Furthermore, to address potential issues, such as low biomass in neonate fecal samples and spurious taxa caused by sequencing errors, we applied the filter_taxa function to filter the ASV data. Only those ASVs present with a minimum count of 2 in at least 10% of fecal samples were retained.

Community composition and alpha diversity indices, including Chao1, Shannon, and Simpson diversity, were calculated using the filtered ASV table and the microbiome package [57]. Differences in the alpha diversity indices were assessed using the Mann–Whitney U test. To further address taxonomic differences in the gut microbiota, the abundant microbial communities were identified using the analysis of compositions of microbiomes with bias correction (ANCOM-BC) (v1.2.2) [58]. Adjustments were made for confounding factors, and correction values obtained from the models were adjusted using the Bonferroni method (*q* < 0.05). Taxa whose proportion of zeroes exceeded 90% were excluded from the analysis. Beta diversity analysis was conducted using the vegan package [59], and PERMANOVA with 9999 permutations was performed to investigate the impact of antibiotics and probiotics on the gut microbiota community. To achieve the visualization of beta diversity, we carried out principal coordinate analysis (PCoA) using the vegan and ggplot2 packages [59,60]. Furthermore, we identified the core genera that were consistently present in the majority of infant fecal samples; this was achieved using the core_members function of the microbiome package. To determine the core genera, a threshold of a 0.01% presence in 75% of infant fecal samples was applied, and the result was transformed compositionally and visualized using ggplot2.

The raw data of antibiotic resistance genes were preprocessed and subjected to logarithmic transformation. Due to the three duplications of each sample, an average value of the sample copies was calculated before the transformation so that a better conformity to the assumption of normal distribution could be obtained. Then, the processed sample data were subjected to statistical analysis, including *t*-tests, analysis of variance (ANOVA), and Spearman correlation analysis with microbial communities.

## 5. Conclusions

This study demonstrates that in the first six months of life, exposure to antibiotics and probiotics significantly impacts the composition and structure of the infant’s gut microbiota. Antibiotics decrease the abundance of key taxa and increase the prevalence of ARGs, while probiotic intake does not exhibit such effects. Importantly, certain dominant bacterial taxa, such as *Enterococcus* and *Escherichia*, were identified as potential reservoirs for ARGs in the infant gut. This study highlights the critical need to optimize antibiotic and probiotic use in early life to protect the infant’ gut microbiome and mitigate antimicrobial resistance, with the expectation that further research on the gut microbiome–resistance relationship will guide evidence-based strategies to promote lifelong health through preserving a healthy infant gut ecosystem.

## Figures and Tables

**Table 1:** Sociodemographic characteristics of mothers and infants in the first three days, at two months, and at six months.

Variable		First 3d	2 m	6 m
*n* = 189		77	55	57
Mother				
Age (Year)	Mean ± SD	27.4 ± 3.6	26.8 ± 3.6	27.6 ± 3.3
	<25	21 (27.3)	20 (36.4)	12 (21.1)
	≥25	56 (72.7)	35 (63.6)	45 (78.9)
Education (Year) ^a^	Mean ± SD	10.6 ± 2.7	10.7 ± 2.7	10.7 ± 3.1
	<9	42 (55.3)	30 (55.6)	32 (57.1)
	≥9	34 (44.7)	24 (44.4)	24 (42.9)
Occupation ^b^				
	Farmer	59 (77.6)	41 (75.9)	44 (78.6)
	Non-farmer	17 (22.4)	13 (24.1)	12 (21.4)
Height (m)	Mean ± SD	1.6 ± 0.05	1.6 ± 0.05	1.6 ± 0.05
Weight (kg)	Mean ± SD	56.0 ± 7.7	55.1 ± 7.5	54.4 ± 8.0
Pre-pregnancy BMI (kg/m^2^)	Mean ± SD	21.8 ± 3.2	21.4 ± 2.9	21.4 ± 3.2
Delivery mode				
	VD	54 (70.1)	42 (76.4)	41 (71.9)
	CS	23 (29.9)	13 (23.6)	16 (28.1)
Infant				
Gender				
	Male	36 (46.7)	22 (40.0)	26 (45.6)
	Female	41 (53.3)	33 (60.0)	31 (54.4)
Gestational age (Week)	Median (P25, P75)	39.2 (39.0, 40.1)	39.2 (39.0, 40.1)	39.2 (39.0, 40.1)
Birth weight (g)	Mean ± SD	3230.5 ± 386.8	3183.6 ± 411.5	3186.0 ± 377.3
Birth length (cm) ^c^	Mean ± SD	51.6 ± 1.8	51.6 ± 2.0	51.3 ± 1.7
IAP				
	Yes	18 (23.4)		
	No	59 (76.6)		
Only antibiotics (ABX)			4 (7.3)	5 (8.8)
Only probiotics (PRO)			9 (16.4)	9 (15.8)
Both antibiotics and probiotics (ABX + PRO)			2 (3.6)	0
Neither antibiotics nor probiotics (CON)			40 (72.7)	43 (75.4)

^a^ “Education” data missing (*n* = 1); ^b^ “Occupation” data missing (*n* = 1); ^c^ “Birth length” data missing (*n* = 1); VD, vaginal delivery; CS, cesarean section; IAP, intrapartum antibiotic prophylaxis; ABX, use only antibiotics; PRO, use only probiotics; ABX + PRO, use both antibiotics and probiotics; CON, use neither antibiotics nor probiotics.

**Table 2:** Alpha diversity of infant gut microbiota in the first three days, median (P25, P75).

α Diversity Indices	IAP (*n* = 18)	CON (*n* = 59)	*p* Value
Chao1			
General	35.5 (23.0, 80.0)	60.0 (30.0, 86.0)	0.233
VD	52.0 (26.0, 89.0)	58.0 (30.0, 85.0)	0.871
CS	25.0 (23.0, 57.0)	67.5 (40.0, 95.0)	0.186
Shannon			
General	1.8 (0.9, 2.3)	1.7 (0.8, 3.0)	0.895
VD	2.2 (1.1, 2.3)	1.5 (0.7, 2.5)	0.494
CS	1.8 (0.5, 2.2)	2.0 (1.2, 3.3)	0.208
Simpson			
General	0.8 (0.4, 0.8)	0.7 (0.3, 0.9)	0.914
VD	0.8 (0.6, 0.8)	0.6 (0.3, 0.8)	0.523
CS	0.7 (0.2, 0.8)	0.8 (0.5, 0.9)	0.186

**Table 3:** Alpha diversity of infant gut microbiota at two months and six months of age, median (P25, P75).

α Diversity Indices	ABX	PRO	ABX + PRO	CON	*p* Value ^a^
2m (*n* = 55)	4	9	2	40	
Chao1					
General	85.5 (57.5, 90.0)	55.0 (34.0, 87.0)	43.5 (40.0, 47.0)	42.5 (34.0, 73.5)	0.405
VD	83.0 (32.0, 88.0)	47.0 (34.0, 87.0)	43.5 (40.0, 47.0)	43.0 (34.0, 73.0)	0.793
CS	92.0 (92.0, 92.0)	84.0 (28.0, 89.0)	-	39.0 (34.0, 77.0)	0.242
Shannon					
General	2.2 (2.1, 2.2)	2.4 (1.7, 2.5)	2.0 (1.8, 2.2)	1.9 (1.5, 2.3)	0.484
VD	2.1 (2.1, 2.2)	2.5 (2.2, 2.6)	2.0 (1.8, 2.2)	1.9 (1.5, 2.3)	0.309
CS	2.2 (2.2, 2.2)	1.7 (1.2, 2.4)	-	1.6 (1.2, 2.3)	0.724
Simpson					
General	0.8 (0.8, 0.8)	0.9 (0.7, 0.9)	0.8 (0.7, 0.8)	0.8 (0.6, 0.8)	0.426
VD	0.8 (0.8, 0.8)	0.9 (0.9, 0.9)	0.8 (0.7, 0.8)	0.8 (0.7, 0.8)	0.239
CS	0.8 (0.8, 0.8)	0.7 (0.6, 0.9)	-	0.6 (0.4, 0.8)	0.724
6m (*n* = 57)	5	9	0	43	
Chao1					
General	85.0 (65.0, 90.0)	59.0 (40.0, 74.0)	-	81.0 (44.0, 93.0)	0.345
VD	75.0 (50.5, 87.5)	74.0 (62.0, 91.0)	-	79.0 (44.0, 91.0)	0.807
CS	91.0 (91.0, 91.0)	34.5 (26.0, 42.5)	-	82.0 (79.0, 101.0)	0.049
Shannon					
General	1.7 (1.6, 2.0)	1.8 (1.7, 2.3)	-	2.0 (1.7, 2.4)	0.428
VD	1.7 (1.1, 1.9)	1.8 (1.7, 2.7)	-	2.0 (1.7, 2.4)	0.286
CS	2.1 (2.1, 2.1)	1.8 (1.3, 2.1)	-	2.0 (1.7, 2.2)	0.726
Simpson					
General	0.6 (0.6, 0.7)	0.7 (0.7, 0.8)	-	0.8 (0.7, 0.8)	0.261
VD	0.6 (0.4, 0.7)	0.7 (0.7, 0.9)	-	0.8 (0.7, 0.8)	0.130
CS	0.8 (0.8, 0.8)	0.8 (0.5, 0.8)	-	0.7 (0.6, 0.8)	0.820

^a^ The *p*-values were derived from nonparametric comparisons between the four groups.

**Table 4:** Core genus abundance of infant gut microbiota in the first three days of age, median (P25, P75).

Core Genus	General (*n* = 77)	IAP (*n* = 18)	CON (*n* = 59)	*p* Value ^a^
*ASV1: Escherichia. Escherichia_coli*				
General	50.1 (13.2, 91.3)	26.6 (2.1, 88.8)	52.5 (23, 92)	0.102
VD	66.8 (26.5, 96.7)	56.7 (13.3, 91.3)	69.3 (31.4, 97.1)	0.372
CS	22.5 (0.4, 50.1)	15.3 (2.1, 33.6)	32.9 (0.4, 50.1)	0.659
*ASV2: Bifidobacterium. Bifidobacterium_pseudocatenulatum*				
General	1.6 (0.2, 13.9)	0.2 (0, 3.7)	4.2 (0.6, 17.3)	0.009
VD	2.3 (0.4, 11)	0.3 (0, 4.5)	3.6 (0.6, 11)	0.147
CS	1 (0, 14.8)	0 (0, 3.6)	10.6 (0.3, 18.3)	0.035
*ASV3: Bifidobacterium. Bifidobacterium_longum*				
General	1.5 (0.1, 13.5)	0.5 (0, 17.3)	2.2 (0.1, 13.5)	0.709
VD	1.5 (0.2, 13.4)	0.5 (0.2, 18.2)	2.1 (0.3, 12.4)	0.991
CS	1.5 (0.1, 17.2)	0.5 (0, 9.5)	5.9 (0.1, 17.2)	0.571
*ASV6: Enterococcus. Enterococcus_faecium*				
General	1.2 (0.1, 6.1)	2.3 (0.5, 8.2)	0.7 (0, 6.1)	0.152
VD	0.3 (0, 3.6)	1.2 (0.5, 5.5)	0.3 (0, 2.8)	0.104
CS	6.9 (1.2, 88.7)	3.4 (0.8, 8.2)	13.3 (3.8, 88.7)	0.314
*ASV7: Streptococcus. Streptococcus_salivarius*				
General	1 (0, 5.1)	1.3 (0.1, 6.5)	0.4 (0, 4.9)	0.229
VD	0.4 (0, 2.9)	1.2 (0.1, 5.9)	0.4 (0, 2.2)	0.384
CS	3.2 (0.1, 8.7)	1.4 (0.2, 6.5)	4 (0, 8.7)	0.801
*ASV30: Staphylococcus. Staphylococcus_epidermidis*				
General	0.3 (0, 1.7)	0.5 (0, 4.6)	0.2 (0, 1.7)	0.596
VD	0.1 (0, 1.7)	0.3 (0.1, 1.4)	0.1 (0, 1.7)	0.935
CS	0.7 (0, 29.5)	1.6 (0, 34.3)	0.5 (0.1, 1.7)	0.488

^a^ The *p*-values were derived from nonparametric comparisons between the IAP and CON groups.

**Table 5:** Core genus abundance of infant gut microbiota at two months of age, median (P25, P75) ^a^.

Core Genus	General (*n* = 55)	ABX (*n* = 4)	PRO (*n* = 9)	CON (*n* = 40)	*p* Value ^b^
*ASV1: Escherichia. Escherichia_coli*					
General	8.8 (1.7, 26.2)	7.6 (4.5, 23)	7.1 (5.7, 13.4)	11.7 (2.4, 35.7)	0.168
VD	10 (2.7, 34.1)	7.3 (1.7, 38)	6.7 (5.7, 13.4)	13.7 (5.5, 41)	0.123
CS	7.1 (1.6, 15)	8 (8, 8)	7.1 (1.6, 15)	2.2 (1.1, 17.2)	0.943
*ASV2: Bifidobacterium. Bifidobacterium_pseudocatenulatum*					
General	1.8 (0.2, 11.2)	13 (7.7, 32.1)	17.1 (1.1, 33.1)	1.4 (0.2, 6.5)	0.064
VD	1.8 (0.4, 10.6)	10.6 (4.8, 48.6)	25.1 (1.2, 41.3)	1.4 (0.4, 6.7)	0.057
CS	1.1 (0.2, 15.5)	15.5 (15.5, 15.5)	1.1 (0, 33.1)	0.6 (0.2, 5.7)	0.709
*ASV3: Bifidobacterium. Bifidobacterium_longum*					
General	8.7 (1.7, 27)	31.9 (15.9, 38)	9.4 (1.4, 19)	7.4 (1.5, 27)	0.429
VD	11.8 (3.8, 28.3)	29.9 (1.9, 33.8)	14.2 (1.9, 24.6)	10.8 (3.8, 29.8)	0.855
CS	2.2 (1, 13.3)	42.2 (42.2, 42.2)	1.4 (1.1, 13.3)	2.2 (0, 8.7)	0.268
*ASV4: Bifidobacterium. Bifidobacterium_longum*					
General	2.2 (1, 10.5)	0.7 (0.3, 0.9)	1.7 (1.2, 1.9)	3.3 (1.3, 22.4)	0.072
VD	1.9 (0.8, 8.8)	0.6 (0, 1)	1.3 (0, 1.9)	2.6 (1.1, 20.8)	0.121
CS	4.1 (1.8, 70.5)	0.8 (0.8, 0.8)	1.8 (1.7, 73.1)	4.3 (3.1, 70.5)	0.314
*ASV5: Bifidobacterium. Bifidobacterium_breve*					
General	1.7 (0.5, 13.2)	18.2 (6.3, 53.1)	1.7 (0.1, 2.5)	1.4 (0.5, 12.4)	0.120
VD	2.3 (0.3, 13.6)	12 (0.6, 81.8)	0.9 (0, 1.9)	2.7 (0.3, 13.6)	0.150
CS	1.4 (0.9, 2.5)	24.4 (24.4, 24.4)	2.5 (1.3, 2.5)	1.1 (0.9, 1.6)	0.273
*ASV6: Enterococcus. Enterococcus_faecium*					
General	0.8 (0.2, 2.8)	4.6 (1.9, 9.2)	4.8 (1.1, 14.3)	0.5 (0.1, 1.5)	0.008
VD	0.8 (0.2, 2.1)	2.8 (1.1, 11.9)	3.5 (1.1, 9.7)	0.5 (0.1, 1.3)	0.019
CS	1.3 (0.5, 14.3)	6.5 (6.5, 6.5)	14.3 (0.7, 18.9)	0.5 (0.2, 4.7)	0.380
*ASV7: Streptococcus. Streptococcus_salivarius*					
General	3.1 (1.1, 9)	0.9 (0.4, 1.7)	2.7 (1.3, 6.8)	3.7 (1.2, 13.6)	0.117
VD	3.1 (1.3, 9)	1.4 (0.3, 2.1)	4.7 (1.3, 8.5)	3.5 (1.3, 13.1)	0.228
CS	1.6 (0.6, 8.9)	0.4 (0.4, 0.4)	1.6 (0.3, 3.1)	3.9 (0.6, 22.7)	0.392
*ASV13: Bifidobacterium. Bifidobacterium_bifidum*					
General	1.9 (0.4, 6.7)	1.4 (0.5, 3.8)	1.9 (0.8, 14.4)	2.2 (0.4, 6.4)	0.737
VD	2 (0.5, 6.9)	0.9 (0, 5.6)	2.8 (1.8, 14.4)	2.1 (0.4, 6.9)	0.652
CS	1.9 (0.4, 3)	1.9 (1.9, 1.9)	0.8 (0.2, 14.8)	2.3 (0.4, 3)	0.999
*ASV18: Ruminococcus. Ruminococcus_gnavus*					
General	0.3 (0.1, 1.3)	0.2 (0.1, 0.6)	0.2 (0.1, 0.9)	0.3 (0.1, 1.7)	0.834
VD	0.5 (0.1, 1.6)	0.1 (0.1, 0.9)	0.4 (0, 0.9)	0.5 (0.1, 2.3)	0.658
CS	0.1 (0.1, 0.3)	0.3 (0.3, 0.3)	0.1 (0.1, 88.1)	0.1 (0, 0.3)	0.590
*ASV34: Erysipelatoclostridium. Erysipelatoclostridium_ramosum*					
General	0.1 (0, 0.5)	0.1 (0, 0.1)	0.1 (0.1, 0.3)	0.1 (0, 0.6)	0.781
VD	0.1 (0, 0.6)	0.1 (0, 0.1)	0.3 (0.1, 1.8)	0.1 (0, 0.6)	0.456
CS	0 (0, 0)	0.1 (0.1, 0.1)	0.1 (0, 0.1)	0 (0, 0.2)	0.693

^a^ The group that uses both antibiotics and probiotics (ABX + PRO group) is not shown due to the small sample size (*n* = 2). ^b^ The *p*-values were derived from nonparametric comparisons between the three groups.

**Table 6:** Core genus abundance of infant gut microbiota at six months of age, median (P25, P75).

Core Genus	General (*n* = 57)	ABX (*n* = 5)	PRO (*n* = 9)	CON (*n* = 43)	*p* Value ^a^
*ASV1: Escherichia. Escherichia_coli*					
General	9.6 (2.4, 23.8)	15.8 (1.5, 23.8)	12.6 (4.3, 26.9)	8.2 (2.4, 21.8)	0.911
VD	6.3 (2, 21.8)	12.6 (0.9, 45.2)	17.4 (12.3, 26.9)	5.1 (2, 17.2)	0.336
CS	13.9 (6.2, 28.6)	15.8 (15.8, 15.8)	7.2 (1.2, 20.5)	15.2 (8, 30)	0.474
*ASV2: Bifidobacterium. Bifidobacterium_pseudocatenulatum*					
General	1.3 (0.7, 10.8)	1.1 (0.7, 1.2)	0.7 (0, 1)	2.4 (0.8, 22.4)	0.004
VD	2.4 (1, 19.6)	1.2 (0.5, 1.8)	1 (1, 1)	5.5 (1.1, 26.9)	0.048
CS	0.8 (0.3, 1.5)	0.7 (0.7, 0.7)	0 (0, 0.3)	1.3 (0.7, 1.8)	0.041
*ASV3: Bifidobacterium. Bifidobacterium_longum*					
General	6.7 (1.4, 19.3)	1.8 (1.6, 6.7)	1.2 (0.8, 7.1)	8.8 (1.7, 19.5)	0.125
VD	6.9 (1.6, 19.5)	1.7 (1.1, 4.2)	1 (0.8, 7.1)	9.6 (2.2, 22.5)	0.114
CS	2.1 (1.1, 15)	7.7 (7.7, 7.7)	1.4 (0.6, 19.5)	2.5 (1, 19.3)	0.717
*ASV4: Bifidobacterium. Bifidobacterium_longum*					
General	3 (0.9, 52.8)	0.6 (0.2, 13)	52.8 (1.6, 60)	2.7 (1, 48)	0.393
VD	1.8 (0.4, 28.8)	0.4 (0.1, 31)	58.3 (4.2, 60)	1.8 (0.4, 13.9)	0.197
CS	31.6 (2.1, 60.7)	13 (13, 13)	27.2 (0.8, 69.9)	48 (2.7, 68.1)	0.870
*ASV5: Bifidobacterium. Bifidobacterium_breve*					
General	1.7 (0.7, 14.1)	1.5 (1.1, 52.6)	0.5 (0.2, 0.9)	2.1 (0.9, 14.8)	0.106
VD	1.8 (0.8, 19.4)	1.3 (0.8, 39.9)	0.5 (0.4, 0.9)	4.3 (0.9, 31.1)	0.276
CS	1.6 (0.6, 11.4)	52.6 (52.6, 52.6)	0.4 (0.1, 26.9)	1.6 (1.2, 10)	0.221
*ASV6: Enterococcus. Enterococcus_faecium*					
General	1 (0.2, 2.9)	0.4 (0.4, 24.4)	1 (0.2, 1.8)	1.1 (0.2, 2.9)	0.938
VD	0.6 (0.2, 2.5)	12.4 (0.2, 59.7)	0.4 (0.1, 1.8)	0.7 (0.2, 2.4)	0.701
CS	1.1 (0.3, 3.5)	0.4 (0.4, 0.4)	1 (0.6, 27.3)	1.9 (0.2, 3.6)	0.750
*ASV7: Streptococcus. Streptococcus_salivarius*					
General	1 (0.3, 3.2)	0.5 (0.4, 2.3)	3.1 (0.7, 4.5)	0.9 (0.3, 2.2)	0.655
VD	0.6 (0.3, 2.2)	0.5 (0.3, 4)	3.1 (0.3, 5.3)	0.9 (0.3, 2)	0.804
CS	1.5 (0.4, 4.3)	2.3 (2.3, 2.3)	2.5 (0.8, 4.3)	0.9 (0.3, 8.4)	0.762
*ASV9: Blautia. Blautia_obeum*					
General	0.3 (0.1, 0.9)	0.3 (0.2, 0.3)	0.2 (0, 0.3)	0.6 (0.2, 1.6)	0.013
VD	0.6 (0.2, 1.4)	0.3 (0.1, 0.6)	0.3 (0.2, 0.3)	0.7 (0.2, 2.6)	0.161
CS	0.2 (0, 0.4)	0.2 (0.2, 0.2)	0 (0, 0)	0.3 (0.1, 0.5)	0.047
*ASV12: Collinsella. Collinsella_aerofaciens*					
General	0.1 (0.1, 0.2)	0.1 (0.1, 0.1)	0.2 (0, 0.2)	0.2 (0.1, 0.3)	0.684
VD	0.2 (0, 0.9)	0.1 (0, 0.5)	0.2 (0.2, 0.2)	0.2 (0.1, 1.4)	0.644
CS	0.07 (0, 0.1)	0.1 (0.1, 0.1)	0 (0, 0.1)	0.1 (0.1, 0.1)	0.275
*ASV13: Bifidobacterium. Bifidobacterium_bifidum*					
General	2 (0.2, 4.5)	0.2 (0.1, 1.8)	0.6 (0.2, 2)	2.4 (0.3, 5.9)	0.217
VD	1.8 (0.2, 4.2)	0.2 (0, 1)	0.3 (0.2, 0.8)	2.2 (0.3, 5.8)	0.082
CS	2.5 (0.5, 6.1)	6.4 (6.4, 6.4)	1.3 (0.3, 6.3)	2.6 (0.4, 6.1)	0.447
*ASV18: Ruminococcus. Ruminococcus_gnavus*					
General	0.3 (0.1, 1.6)	0.2 (0.1, 0.2)	0.1 (0, 0.9)	0.5 (0.2, 2)	0.256
VD	0.3 (0.1, 2)	0.2 (0.1, 3.8)	0.3 (0.1, 0.9)	0.5 (0.1, 2.1)	0.876
CS	0.3 (0.1, 1.2)	0.2 (0.2, 0.2)	0 (0, 0.6)	0.5 (0.2, 1.6)	0.178
*ASV19: Akkermansia. Akkermansia_muciniphila*					
General	0.2 (0, 0.5)	0.2 (0.1, 0.2)	0.1 (0, 0.2)	0.2 (0, 0.5)	0.682
VD	0.2 (0, 0.5)	0.2 (0, 0.3)	0.2 (0.1, 0.2)	0.2 (0, 0.6)	0.869
CS	0.2 (0, 6.4)	0.2 (0.2, 0.2)	0 (0, 16)	0.2 (0.1, 0.4)	0.531
*ASV34: Erysipelatoclostridium. Erysipelatoclostridium_ramosum*					
General	0.1 (0, 0.8)	0.1 (0, 0.1)	0 (0, 0.1)	0.2 (0.1, 1.1)	0.026
VD	0.2 (0, 1.1)	0.1 (0, 3.4)	0 (0, 0.1)	0.2 (0.1, 1.3)	0.276
CS	0 (0, 0.3)	0.1 (0.1, 0.1)	0 (0, 0)	0.1 (0.1, 0.8)	0.117
*ASV39: Streptococcus. Streptococcus_oralis*					
General	0.1 (0, 0.1)	0.1 (0.1, 0.1)	0.1 (0, 0.2)	0.1 (0, 0.1)	0.596
VD	0 (0, 0.1)	0.1 (0, 0.1)	0.1 (0.1, 0.1)	0.1 (0, 0.1)	0.696
CS	0 (0, 0.1)	0.2 (0.2, 0.2)	0.1 (0, 3.8)	0 (0, 0.1)	0.376

^a^ The *p*-values were derived from nonparametric comparisons between the three groups.

## Data Availability

The data that support the findings of this study are available upon request from the corresponding author upon reasonable request.

## Supplementary Materials

The following supporting information can be downloaded at https://www.mdpi.com/article/10.3390/antibiotics13070602/s1, Figure S1: Relative abundance of gut microbial communities at phylum level and genus level at two months (A,B) and at six months of age (C,D); Table S1: ANCOM-BC analysis of different bacterial genera in infant fecal samples with and without IAP use stratified by delivery mode; Table S2: PERMANOVA analysis of beta diversity in infant fecal samples with antibiotics/probiotics in the first three days, at two months, and at six months; Table S3: Absolute abundance of six antibiotic resistance genes in infant fecal samples in the first three days, at two months, and at six months (log copies/µL) (log copies/µL); Table S4: Spearman correlation result of ARGs and microbial communities in the first three days; Table S5: Spearman correlation result of ARGs and microbial communities at two months of age; Table S6: Spearman correlation result of ARGs and microbial communities at six months of age.
