# Supplementary material for: Unraveling the Microbial Symphony: Impact of Antibiotics and Probiotics on Infant Gut Ecology and Antibiotic Resistance in the First Six Months of Life
Source: Antibiotics (Basel). 2024 Jun 27;13(7):602. doi: 10.3390/antibiotics13070602 (PMC11274100; doi:10.3390/antibiotics13070602)
Supplement: Supplementary file 1 [file antibiotics-13-00602-s001.zip › antibiotics-2991493-Supplementary Figure.pdf]

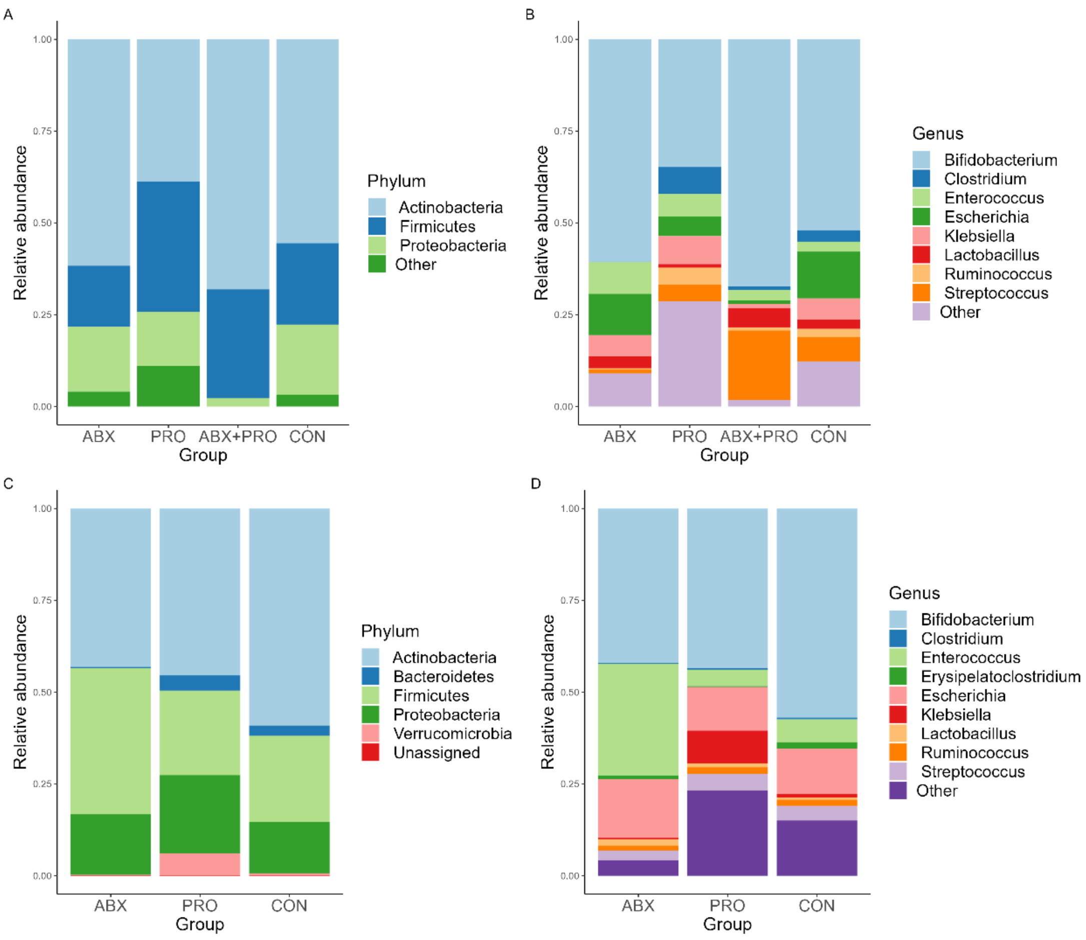

Figure S1. Relative abundance of gut microbial communities at phylum level and genus level at two months (**A**,**B**) and at six months of age (**C**,**D**). ABX, use only antibiotics; PRO, use only probiotics; CON, use neither antibiotics nor probiotics.
